# Precision genome editing in plants: state-of-the-art in CRISPR/Cas9-based genome engineering

**DOI:** 10.1186/s12870-020-02385-5

**Published:** 2020-05-25

**Authors:** Naoki Wada, Risa Ueta, Yuriko Osakabe, Keishi Osakabe

**Affiliations:** Graduate School of Technology, Industrial and Social Sciences, Tokushima University, Tokushima, Japan

**Keywords:** Plant genome engineering, Null segregant, CRISPR/Cas9, CRISPR/dCas9

## Abstract

Traditionally, generation of new plants with improved or desirable features has relied on laborious and time-consuming breeding techniques. Genome-editing technologies have led to a new era of genome engineering, enabling an effective, precise, and rapid engineering of the plant genomes. Clustered regularly interspaced short palindromic repeats (CRISPR)/CRISPR-associated protein 9 (CRISPR/Cas9) has emerged as a new genome-editing tool, extensively applied in various organisms, including plants. The use of CRISPR/Cas9 allows generating transgene-free genome-edited plants (“null segregants”) in a short period of time. In this review, we provide a critical overview of the recent advances in CRISPR/Cas9 derived technologies for inducing mutations at target sites in the genome and controlling the expression of target genes. We highlight the major breakthroughs in applying CRISPR/Cas9 to plant engineering, and challenges toward the production of null segregants. We also provide an update on the efforts of engineering Cas9 proteins, newly discovered Cas9 variants, and novel CRISPR/Cas systems for use in plants. The application of CRISPR/Cas9 and related technologies in plant engineering will not only facilitate molecular breeding of crop plants but also accelerate progress in basic research.

## Background

Production of plants with improved traits drives the current reliance of agriculture and various industries on plant resources. Traditionally the plant breeding has been done by crossing and selection. However, the traditional breeding methods are labor- and time-intensive. Genome editing allows targeting and modifying specific DNA sequences [1–4]. Generally, introducing mutations in the target DNA sequence using genome-editing technologies involves three common steps. First, an exogenous engineered nuclease consisting of recognition module and nuclease domain recognizes the target DNA sequence. Then, the engineered nuclease binds to the target DNA sequence and induces double-strand breaks (DSBs) at or in the vicinity of the target site. The endogenous nonhomologous end-joining (NHEJ) or homology-directed repair (HDR) pathways then repair the DSB. While NHEJ is an error-prone repair process and often results in the introduction of mutations, such as small insertions and deletions (Indel), HDR results in a precise repair of DSBs [1, 2]. These technologies have been successfully applied in various organisms, including plants [1–3].

Three technologies have been developed as major genome-editing technologies [1–3]. At first, zinc finger nuclease (ZFN) has been reported as an engineered nuclease. Secondary, transcription activator-like effector nuclease (TALEN) has appeared as a more flexible engineered nuclease. Finally, the clustered regularly interspaced short palindromic repeat (CRISPR)/CRISPR-associated protein 9 nuclease (Cas9) has been developed as a more simple and flexible engineered nuclease. ZFNs and TALENs consist of a sequence-specific DNA binding module and a FokI nuclease domain. The FokI nuclease domain requires dimerization to become an active nuclease. Therefore, two modules need to be designed to target closely spaced DNA sequences, allowing dimerization of FokI at the target DNA sequence. This requirement for dimerization gives specificity to ZFNs and TALENs. However, in fact, it is expensive and difficult to design active nucleases [1, 3]. By contrast, the CRISPR/Cas9 system is inexpensive, and the experimental design is easy [1–3]. The CRISPR/Cas9 system emerged as a genome-editing tool in 2012 [4–6]. The simplicity, ease, and high efficiency of the CRISPR/Cas9 system have facilitated its development into the most widely applied genome-editing tool.

The CRISPR/Cas9 system has two major components: Cas9 protein and guide RNA (gRNA). Cas9 protein is an RNA-dependent DNA endonuclease that forms a complex with gRNA. The gRNA is a small RNA that contains 20 nt nucleotides complementary to target sequences and is necessary for recruiting Cas9 protein to the target site. This is the main difference between CRISPR/Cas9 and the other genome-editing technologies, that is, it relies on the DNA-RNA interaction, instead of DNA-protein interaction, for target DNA sequence recognition. In the case of ZFNs and TALENs that target the specific sequence by DNA–protein interaction, design and expression of two different DNA-binding domains (500–700 amino acid in case of TALEN) are required per target site. This process is rather laborious. On the other hand, in the case of CRISPR/Cas9, which uses DNA–RNA interaction, design of an 18–20 bp oligonucleotide is all that is required. This makes it very easy to adapt the CRISPR/Cas9 system for genome editing applications. To function as a genome editing tool, Cas9 and gRNA have to bind to a specific protospacer adjacent motif (PAM) sequence, which is a short nucleotide sequence located at the 3′ end of the target sequence. In the case of *Streptococcus pyogenes* Cas9 (SpCas9), which is the Cas9 most commonly used for genome editing, the sequence 5′-NGG-3′ is recognized as the PAM. Unless otherwise mentioned, the genome editing experiments presented in the current review have been done using SpCas9.

Recruitment of Cas9 usually results in DSBs at the target site in the genome, but the unintended changes (off-target effects) have sometimes been induced. The CRISPR/Cas9 system has been developed for precise genome editing with minimal effects on the genome by enhancing the specificity or avoiding the DSB [2, 3]. Furthermore, when the desired mutant plants were developed, removal of the exogenous transgenes has been evaluated attentively [3].

In this review, we summarized the recent approaches for the targeted manipulation of plant genome using CRISPR/Cas9, focusing on the ones that lead to heritable genome modifications even in the absence of transgenes and on the production of transgene-free plants (null segregants), discussing their advantages and disadvantages, and scope for further development. We also introduce the newly developed and discovered CRISPR/Cas systems as promising tools for plant genome engineering in the near future.

## Genome editing using CRISPR/Cas9 in plants: an overview

The CRISPR/Cas9 system has been successfully applied in various plant species. These include not only model plants, such as *Arabidopsis*, but also crops, such as rice, tobacco, sorghum, wheat, maize, soybean, tomato, potato, poplar, apple and banana [1, 3]. Calli, leaf discs, protoplasts and flowers have been used as a plant material. The purpose of the applications includes the enhancement of abiotic or biotic stress resistance, engineering of metabolic pathways and increase of grain yield. The introduced mutations are inherited by the next generation of plants, indicating that plant genome editing can be used for plant research and the production of useful plants.

An important advantage of using the CRISPR/Cas9 system is the possibility of simultaneously editing multiple target genes [7–9]. For example, Zsögön et al., [7] has targeted six genes in a two-step experimental approach and induced mutations into four genes. By applying the second round of genome editing experiments, the simultaneous genome editing of six genes were achieved. This study is also important because they have achieved the de novo domestication of wild tomato by targeting the six loci important for key domestication traits. The results indicates that multiplex genome editing using CRISPR/Cas9 can be used to mimic the domestication process during evolution in a short time frame, with implications for a rapid and convenient generation of new plants with desirable traits. Simultaneous targeting of multiple sites also can induce deletions with defined sizes between target sites [8, 9], which would be useful for the disruption of regulatory sequences and production of knockout mutants whose gene functions were disrupted not by out-of-frame mutations but by the deletion of a certain region.

Gene targeting (GT) by CRISPR/Cas9 is another approach for engineering the plant genome precisely. GT can be performed via HDR pathway but the HDR efficiency is much lower than NHEJ in plant cells. To enhance GT efficiency in plants, several approaches have been developed, such as suppressing the NHEJ pathway [10, 11], amplifying donor DNAs using virus replicons [12], and driving Cas9 expression using egg-specific promoters [13]. Recently, Miki et al. [13] reported a sequential transformation of *Arabidopsis* for enhanced GT efficiency. The authors first generated parental lines expressing the *Cas9* gene under the control of the egg cell- and early embryo-specific *DD45* promoter from *Arabidopsis*. They then introduced the donor DNA fragment and gRNA expression vectors into selected parental plants that exhibited high genome-editing activity, by the floral dip method. In this manner, the authors achieved heritable gene targeting with 5–10% efficiency (according to the number of T_2_*Arabidopsis* populations examined). However, further improvements in GT efficiency are needed to increase the efficiency of predictable and precise genome editing.

Despite the various benefits of using CRISPR/Cas9, one of the important associated concerns are off-target effects, i.e., unintended mutations at unintended sites induced by genome editing. Several methods have been developed to detect the off-target mutations in vitro and in vivo. These include SITE-seq [14], Digenome-seq [15], CIRCLE-seq [16], GUIDE-seq [17], and DISCOVER-seq [18]. In parallel, the engineering of Cas9 proteins has been performed to enhance the specificity. The details will be discussed later. On the other hand, new types of mutations, which have not been addressed so far, were recently reported. Kosicki et al. [19] observed unexpected large deletions (up to 9.5 kb) elicited as a result of Cas9-based genome editing in mammalian cells. Although such unexpected large deletions have not yet been reported in plants, the possibility of their occurrence should be taken into account.

## Genome manipulation by CRISPR/Cas9 without DSB induction or alteration of the primary genetic material in plants

DSBs are key events in genome editing, but they carry the risk of genome instability and unpredictable outcomes of DNA repair. Therefore, approaches to alter the targeted DNA or gene expression without inducing DSBs have been explored. The key proteins in these approaches are a catalytically dead Cas9 variant (dCas9) that can bind to the target sequence but does not cleave the double-stranded DNA. The dCas9 protein is fused to another effector protein that either modifies the genome or the epigenome without cleaving the double-stranded DNA [2, 20, 21]. Recently, another new approach, called prime editing, has been reported in yeast and mammalian cells [22]. Prime editing can also change DNA information without DSB induction. We introduce this technology briefly in the following section.

### Base editing using dCas9 fused to DNA deaminases

dCas9 proteins fused to DNA deaminases have been developed as base editors that can alter the target DNA sequence without inducing DSBs [23–26]. Cytidine deaminase-incorporating DNA base editors (CBEs; Target-AID and BE) have been developed for nucleotide conversion from C to T, and adenine deaminase-based DNA editor (ABE) has been developed for A to G conversion. Both types of base editors have successfully been used for base editing of the plant genome [3, 27, 28].

Target-AID utilizes PmCDA, a protein from an activation-induced cytidine deaminase (AID) family, fused to dCas9 or Cas9n (D10A) nickase for base editing [24]. By contrast, the BE1, BE2, BE3, and BE4 series utilizes the rat cytidine deaminase rAPOBEC fused to dCas9 or Cas9n (D10A) [23, 26]. The CBE catalyzes the deamination of cytosine to uracil. The uracil is then converted to thymidine by DNA replication or repair processes, resulting in targeted conversion from C to T. Application of CBEs in plant genome engineering has already been reported in *Arabidopsis*, rice, wheat, maize, and tomato [26]. On the other hand, ABE utilizes the adenine DNA deaminase that has been produced by directed evolution of a tRNA adenine deaminase *Escherichia coli* TadA [25]. The ABE catalyzes the deamination of adenosine. The resulting inosine can form a base pair with cytidine, following the incorporation of G in newly synthesized DNA strand. The application of ABE in plant genome engineering has already been reported in *Arabidopsis*, *Brassica napus*, rice, and wheat [26].

**Fig. 1:**
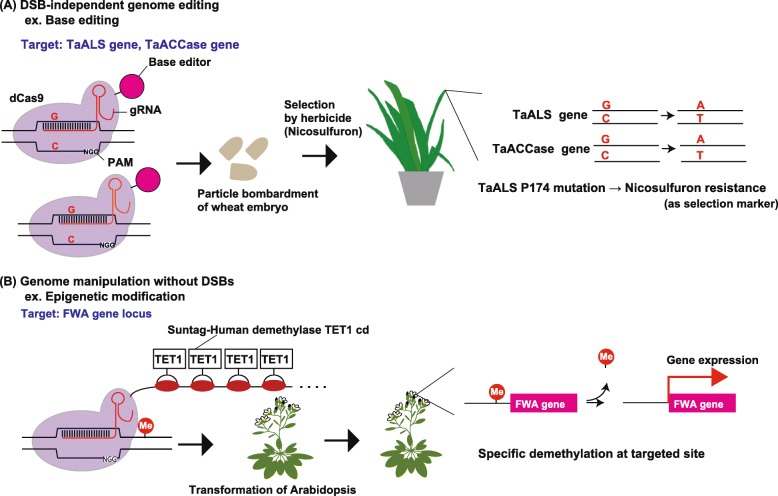
Recent applications of base editing (**a**) and epigenetic editing (**b**) technologies in plants. **a** Co-editing of two genes (*TaALS* and *TaACCase*) by using dCas9 fused with CBE [27]. The approach produced nicosulfuron-resistant wheat plants with mutations in the two genes but without transgenes. **b** Targeted demethylation of the *FWA* promoter by using dCas9-Suntag-hTET1cd in *Arabidopsis* [28]. The approach resulted in the activation of *FWA* gene expression. Demethylation and late-flowering phenotypes were inherited by T2 generation without transgenes

As an example of application of base editing technology into plants, Zhang et al., [27] has developed a co-editing strategy to produce base-edited plants without exogenous selectable markers (Fig. 1a). They found that mutation in the wheat acetolactate synthase (ALS) Pro-174 codon conferred nicosulfuron-herbicide resistance in genome-edited wheat, although the genome-edited plants showed variability in nicosulfuron-resistance. This variability is due to the diversity of the generated genotypes, resulting from the existence of several base-editable sequences within the typical base-editing window (between positions 3 and 9 of the protospacer region) and unexpected mutations outside the base-editing window [27]. In addition, the *ALS* gRNA could target the intragenic alleles in all three subgenomes in wheat, expanding the possibility of genotype variability [27]. They transiently co-expressed the gRNA targeting *TaALS* gene and a gRNA targeting a gene of interest in wheat and selected the genome-edited plants by nicosulfuron resistance. In the case of co-editing of acetyl-coenzyme A carboxylase *(ACCase)* genes, 22–78% of nicosulfuron-resistant plants harbored the mutation in the *ACCase* gene, resulting in the 9–31 fold enrichment of genome-edited plants compared to without nicosulfuron selection. In addition, 33–78% of nicosulfuron-resistant plants lacked transgenes in the T_0_ generation [27]. These results suggested that, although variability in nicosulfuron-resistance was seen, the co-editing strategy could be useful for the production of transgene-free base-edited plants. However, further experimental examples would be needed to demonstrate the utility of the co-editing strategy.

On the other hand, an important question has been raised about the off-target effects of base editing recently. In fact, multiple, in some cases up to tens of thousands, off-target mutations linked to the use of specific BE and ABE editors have been demonstrated in plants [28] and mammalian models [29–31]. Reduction of the off-target effects has been attempted in human cells [29], by using engineered base editors with suppressed RNA-editing activity and increased DNA on-target activity. Further validation will be required for the improvement of the CBE and ABE base-editing systems in plants.

### Targeted epigenetic modifications using dCas9

Epigenetic modifications such as DNA methylation and histone modifications affect the gene expressions. Several examples of transgenerational inheritance of epigenetic status in plants have been reported [32]. Therefore, the targeted epigenetic modifications can be a strategy to cause heritable changes that can be maintained even without transgenes.

The dCas9 protein fused with epigenetic-modifier has been developed as a tool for introducing targeted epigenetic modifications, to alter gene expression in the parental and progeny plants [20]. As an example, Gallego-Bartolomé et al. [33] used dCas9 to introduce heritable targeted DNA demethylation/methylation in plants (Fig. 1b). The authors used a combination of the SunTag system, DNA demethylase TET1cd, and CRISPR/dCas9 (dCas9-SunTag-TET1) to up-regulate the *FLOWERING WAGENINGEN* (*FWA*) gene whose activation causes the late-flower phenotypes in *Arabidopsis*. The dCas9-SunTag-TET1 system composed of a dCas9 fused with GCN peptide repeats and a TET1cd fused with single-chain antibody recognizing GCN peptide. The binding of single-chain antibody to GCN peptide leads to the localized accumulation of TET1cd, resulting in targeted DNA demethylation. They have achieved the demethylation of *FWA* gene promoter region and activation of *FWA* gene expression. The epigenetically edited plants showed the late-flowering phenotypes. The modified epigenetic status and late-flowering phenotypes were stably inherited into next generations even in absence of transgenes.

Targeted epigenetic modification can modify the gene expressions without altering the DNA sequences. However, whether the epigenetic status can be maintained without transgenes in next generations is not clear. Actually Gallego-Bartolomé et al. [33] also targeted another gene, *CACTA1* gene, but the methylation status was recovered after the transgenes were segregated away in next generation. Although it is speculated that the incomplete demethylation attracted the RNA-directed DNA Methylation machinery, the conditions necessary for inheritable epigenetic modifications should be clarified for the further applications in plants.

### Prime editing

Recently, Anzalone et al. [22] have developed a new genome editing technology ‘Prime editing’ in yeast and mammalian cells. They achieved precise genome editing without inducing DSBs or requiring a donor DNA template, which is necessary for genome editing via HDR. Prime editing uses Cas9 nickase fused to reverse transcriptase and engineered gRNA (a prime editing guide RNA, pegRNA), which consists of a primer binding site (PBS), the desired edited sequence, and a sequence that recognizes the target DNA. The Cas9 nickase is recruited to the target DNA sequence by pegRNA, then nicks the PAM-containing DNA strand. The 3′ end of the nicked DNA strand hybridizes to the PBS of pegRNA, priming reverse transcription of the desired edited-sequence on the pegRNA by reverse transcriptase fused to Cas9 nickase. Hybridization between the target DNA and the reverse transcription product produces a 3′ flap with edited-sequence or 5′ flap with unedited-sequence. The 5′ flap is cleaved preferentially by the endonuclease, and the 3′ flap is ligated to the DNA strand. The heteroduplex DNA is repaired by the endogenous DNA repair process, resulting in stable incorporation of the edited sequence into the genome. Prime editing has achieved targeted insertions (up to 44 bp), deletion (up to 80 bp), and all types of point mutations efficiently and precisely. The requirement for an additional two steps of hybridization (target DNA–pegRNA PBS and target DNA–reverse transcript product) for genome editing resulted in much lower off-target editing than with Cas9, which requires only target-DNA–gRNA hybridization. No requirement for DSBs prevented the production of unintended mutations compared to Cas9-initiated HDR. In addition, prime editing has demonstrated more advantages than base editing in cases where multiple cytosines or adenines were present in a base editing window, and bystander edits were unacceptable, because prime editing enabled precise single-nucleotide replacement. The target scope is also expanded in prime editing because, unlike base editing, it is not limited by the need for a PAM sequence at a suitable distance from the target nucleotides. Although prime editing offers advantages compared to other genome editing technologies, it has not yet been applied to plant cells. Prime editing would be a promising technology for plant genome engineering, especially because prime editing can achieve efficient knock-in of DNA fragments in plant cells. Generally, HDR efficiency is low in plant cells, so knock-ins of DNA fragments to target sites is difficult. However, prime editing offers a new strategy for knock-in of DNA fragments via an HDR-independent pathway. It will be interesting to determine if prime editing functions in plant cells as well as in mammalian cells.

## Generation of transgene-free genome-edited plants

**Fig. 2:**
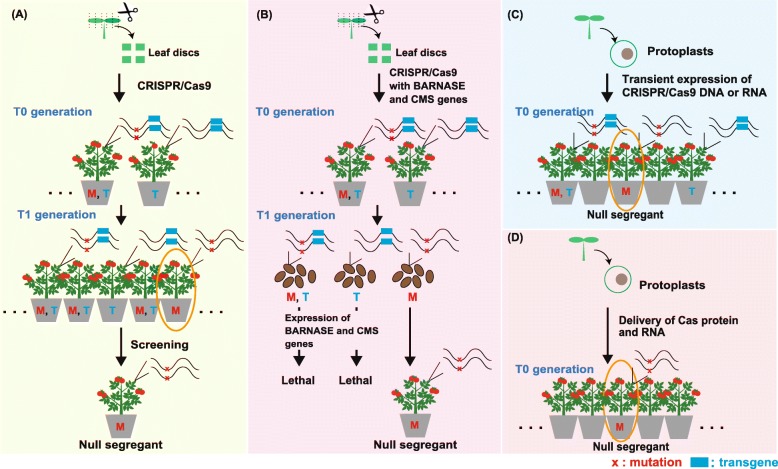
Generation of null segregants in plants by CRISPR/Cas9 technology. Representative methods for the production of null segregants are shown: isolation of null segregants by Mendelian segregation (**a**); programmed self-elimination of transgenic plants (**b**); transient expression of CRISPR/Cas9 (**c**); and ribonucleoprotein-mediated genome editing (**d**). Please refer to the text for detailed explanations. “M” and “T”, plants with mutation and transgene insertion, respectively

Following the revolutionary progress of CRISPR/Cas9-mediated plant genome editing, researchers have focused on the development of efficient approaches to establish genome-edited plants that are transgene-free. The elimination of transgenes contributes to the achievement of precise genome editing, in which unnecessary changes do not exist in the genome. The elimination of the *Cas9* gene from genome-edited plants would also prevent the induction of mutations at untargeted loci. Several tools and approaches, as mentioned later, have been developed so far to reduce off-target effects, although unintended mutations at off-target sites can also be removed by crossing in successive generations in plants. The elimination of transgenes would also alleviate concerns about genome-edited plants. One of the approaches to achieve this purpose is the regeneration of mutant plants without selection pressure. However, this approach is known to be very laborious and time-consuming because the efficiency with which transgene-free mutated plants can be obtained is very low [34]. Therefore, several alternative approaches have been developed. Representative approaches for the production of null segregants are summarized in Table 1 and Fig. 2a–d. These are Mendelian segregation, programmed self-elimination of transgenic plants, transient expression of CRISPR/Cas9, and ribonucleoprotein (RNP)-mediated genome editing. They are described in detail below.

**Table 1:** Methods for the establishment of the null segregants

Methods	Plant materials	Transformation methods	Cas9 and gRNA delivery	Reference
Mendelian segregation (supporeted by new screening strategies)	*Arabidopsis*	*Agrobacterium-mediated transformation (floral dipping)*	Encoding DNA	[35]
	Rice calli	*Agrobacterium-mediated transformation*	Encoding DNA	[34]
Programmed self-elimination of transgenic plants	Rice calli	*Agrobacterium-mediated transformation*	Encoding DNA	[36]
Transient expression of CRISPR/Cas9 from DNA or mRNA	Potato protoplasts	PEG-mediated transformation	Encoding DNA	[37]
	*B. oldhamii, S. italica, O. sativa, Z. mays, A. thaliana, B. oleracea, B. napus, N. tabacum, S. lycopersicum protoplasts*	PEG-mediated transformation	Encoding DNA	[38]
	Tobacco leaf explants	*Agrobacterium-mediated transformation*	Encoding DNA	[39]
	Wheat calli	Particle bombardment	Encoding DNA or RNA	[40]
RNPs-mediated targeted mutagenesis	*Arabidopsis, tobacco, lettuce and rice protoplasts*	PEG-mediated transformation	Protein and in vitro-transcribed gRNA	[41]
	Grape, apple protoplasts	PEG-mediated transformation	Protein and in vitro-transcribed gRNA	[42]
	Petunia × hybrida protolasts	PEG-mediated transformation	Protein and in vitro-transcribed gRNA	[43]
	Wheat protoplast, immmature embryo	PEG-mediated transformation, particle bombardment	Protein and in vitro-transcribed gRNA	[44]
	*B. oleracea and B. rapa protoplasts*	PEG-mediated transformation	Protein and in vitro-transcribed gRNA or synthesized gRNA	[45]
	Maize immmature embryo	Particle bombardment	Protein and in vitro-transcribed gRNA	[46]
	Rice zygote produced by in vitro ferlization of isolated gamates	PEG-mediated transformation	Protein and in vitro-transcribed gRNA	[47]

### Mendelian segregation

The most general approach for obtaining transgene-free plants is to isolate the null segregants by Mendelian segregation (Fig. 2a). This approach usually involves introducing the CRISPR/Cas9 cassettes as DNA and selecting the transgenic plants based on the antibiotic resistance. After the genome-edited plants are identified, the plants are grown until the progenies of the genome-edited plants are obtained. In the progenies, transgenes segregate according to the Mendelian law of segregation when the regenerated plants have no mosaicism. Therefore, there is a possibility that the genome-edited plants whose transgenes segregated away are obtained. Although the screening of genome-edited plants has been usually performed by using PCR, new approaches to facilitate the isolation of null segregants have been developed [34, 35]. For example, Gao et al. [34] employed visual screening of null segregants by expressing the *mCherry* gene from the CRISPR/Cas9 vector. Visual screening for seeds that do not emit the mCherry signals under fluorescence microscope resulted in a quick isolation of the *Cas9*-free *Arabidopsis* mutant among T_2_ seeds. This approach is effective, but nonetheless laborious and time-consuming.

### Programmed self-elimination of transgenic plants

To reduce the time and cost for the selection of progeny in favor of null segregants, programmed self-elimination approach has been used. He et al. [36] utilized two suicide genes (*BARNASE* and *CMS*) for the enrichment and isolation of null segregants in rice (Fig. 2b). BARNASE is a toxic nuclease, and CMS is a rice male gametophyte-specific lethal protein. The authors placed the *BARNASE* gene under the control of the early embryo-specific promoter (*REG2* promoter) and the *CMS* gene under the control of the *35S* promoter in the *Cas9* expression vector, anticipating that transgenic plants harboring the *Cas9* gene and the two suicide genes would be eliminated in a single generation. By applying this strategy, the authors have successfully enriched and isolated null segregants in the T1 generation. However, this strategy can only be used in plants that can be transformed and regenerated by tissue culture, and propagated by seeds. In other words, it cannot be applied to plants that propagate asexually.

### Transient expression of CRISPR/Cas9 from DNA or mRNA

Another approach for obtaining null segregants is to avoid transgene insertion during transformation. This can be achieved by transient expression of CRISPR/Cas9 DNA or mRNA (Fig. 2c). Anderson et al. [37] isolated and transformed protoplasts from potato, and Lin et al. [38] isolated and transformed protoplasts from nine plant species (*Bambusa oldhamii*, *Setaria italica*, *Oryza sativa*, *Zea mays*, *Arabidopsis thaliana*, *Brassica oleracea*, *Brassica napus*, *Nicotiana tabacum*, and *Solanum lycopersicum*). In both studies, mutagenesis was successfully achieved by transformation of protoplasts with CRISPR/Cas9 vectors by PEG treatment. Vector sequences were detected in 10% of the analyzed potato lines [37] and in 17.2% of the genome-edited *N. tabacum* lines [38]. However, the drawback of using protoplasts is that the number of plant species that have established protocols for plant regeneration is limited. As an alternative, *Agrobacterium*-mediated transient expression of the *Cas9* gene has been applied in tobacco leaf discs [39]. Subsequent analysis revealed that 17.2% of genome-edited plants were null segregants. Together, these studies indicate that a transient expression system can be used for the production and isolation of null segregants although it also still carries the risk of transgene integration into the host genome. Therefore, an effective and easy screening system is still needed for the isolation of null segregants.

Zhang et al. [40] compared two strategies, transient expression of the *Cas9* gene, and transient expression of in vitro transcripts (IVT) of *Cas9*-coding sequences in wheat callus cells. The authors used hexaploid bread wheat (*Triticum aestivum* L.) and tetraploid durum wheat (*T. turgidum* L. var. *durum*) calli as plant material, and used particle bombardment as a transformation method. They successfully introduced the mutation in all six alleles in hexaploid bread wheat in the T0 generation. The detectable transgenes were absent in 43.8–86.6% of the T0 mutants. By contrast, no transgenes were detected in T0 mutants obtained via the IVT approach. Considering the above, efficient plant transformation for transient CRISPR/*Cas9* expression is a promising strategy for the establishment of null segregants in the T0 generation.

### RNP-mediated genome editing

DNA-free genome editing is another promising approach for plant genome engineering without transgene integration (Fig. 2d). A ribonucleoprotein (RNP) consisting of Cas9 protein and gRNA can be used for this purpose in case of targeted mutagenesis without donor DNA templates. The RNP can be formed in vitro and transferred into plant protoplasts. Because the RNP does not contain any DNA, transgene integration can be avoided. Woo et al. [41] were the first to demonstrate that the preassembled Cas9-gRNA RNP complex could be directly delivered to plant protoplasts of *Arabidopsis*, tobacco, lettuce, and rice. The authors successfully obtained genome-edited plants with 8.4 to 44% efficiency, and the mutations were stably maintained and transmitted to the progeny. In fact, protoplasts have been used as plant material for the introduction of RNP complexes in several studies [42–45]. The successful application of RNP-mediated genome editing in protoplasts has been reported in grapevine and apple [42], wheat [44], and cabbage and Chinese cabbage [45]. As an alternative, immature embryo transformation using a biolistic method has been used in maize [46] and wheat [44]. In maize, Svitashev et al. [46] reported the efficiency of genome-editing of 47%, when a selection marker was used, and 2.4–9.7% without selection. In wheat, Liang et al. [44] produced mutants with 4.4% efficiency. Importantly, both studies demonstrated a considerable reduction of off-target mutations compared to genome editing by CRISPR/Cas9 DNA. As another example, recently, Toda et al. [47] reported the development of a genome-editing system in which Cas9-gRNA RNP is directly delivered into rice in vitro fertilized zygotes. The genome-editing efficiency reached 4–64% without selection. In addition, Kim et al. [48] demonstrated that purified Cas12a and gRNA could also be used to induce mutations in soybean and wild tobacco.

Collectively, these observations indicate that using the RNP complex might become a prominent strategy of plant genome editing, without transgene integration and with reduced off-target effects. The results also suggest that Cas9 orthologs and variants (see below) could be used for plant genome editing, as RNP, expanding the possibility of plant genome engineering.

## Application of engineered Cas9 and newly discovered Cas proteins for plant genome editing

The applicability of the CRISPR/Cas9 system is limited by the specificity of PAM sequences and the presence of off-target effects. The SpCas9–gRNA complex generally recognizes the region 20 nt upstream of the PAM sequence (5′-NGG-3′). It means that the sequences without NGG can not be selected as a target sequence. Therefore, several approaches have been applied to broaden PAM compatibility and enhance the specificity. These include rational SpCas9 engineering, identification and characterization of Cas9 orthologues and new CRISPR/Cas system from other sources.

The SpCas9 protein is currently extensively engineered to broaden PAM compatibility or to enhance PAM specificity, while reducing off-target effects [49] (Table 2). Rational engineering of SpCas9 proteins based on the crystal structure of Cas9 with gRNA and target DNA has resulted in the generation of engineered Cas9 proteins with different PAM preferences. Kelinstiver et al. [58] reported the generation of SpCas9-VQR, SpCas9-EQR, and SpCas9-VRER with NGA-PAM, NGAG-PAM, and NGCG-PAM, respectively. The SpCas9-VQR, SpCas9-EQR and SpCas9-VRER have also functioned in *Arabidopsis* and rice but their activity were not high when comparing with that of wild-type SpCas9 [59, 60, 62]. Nishimasu et al. [63] developed SpCas9-NG, an SpCas9 with an enhanced compatibility, which recognizes NG-PAM. SpCas9-NG has already been applied in genome editing in plants, for targeted mutagenesis in rice and *Arabidopsis* plants [64–68]. Conversely, SpCas9 proteins with enhanced specificity (SpCas9-HF1 [70], eSpCas9 [72] and HypaCas9 [73]) have also been developed. SpCas9-HF1 and eSpCas9 have been already tested in rice [71]. SpCas9-HF and eSpCas9 show reduced off-target editing activities, suggesting high specificity in plant cells. Finally, directed evolution approaches have been used for Cas9 engineering, resulting in the generation of engineered SpCas9 proteins that show high specificity (xCas9 [76], evoCas9 [74] and Sniper-Cas9 [75]). Expanded PAM preferences have also been detected in xCas9 (NG, GAA, and GAT-PAM). The xCas9 has been tested in *Arabidopsis* and rice [65–68, 77, 78]. Both xCas9 and Cas9-NG could induce mutations at some non-canonical PAMs in plants. However, their efficiency and specificity seem to be different in plant cells. Although Hua et al., [67] reported that xCas9 could work efficiently in rice, other studies [65, 68, 78] reported that xCas9 had much lower activity in rice calli than in mammalian cells [78], and could not recognize NG PAM in tomato [68]. Zhong et al., [65] also reported that xCas9 showed comparable activity to Cas9-WT at NGG PAM, and higher specificity than that of Cas9-WT, but xCas9 activity was not high at the NGH (A, T, C) PAM in rice. On the other hand, Cas9-NG showed higher activity than xCas9 in almost all NG PAM sites in rice [65]. Hua et al. [67] also indicated that Cas9-NG had robust editing activity at several NG PAM sites tested (CGG, AGC, TGA, CGT). These studies suggest that Cas9-NG would be more suitable for genome editing at the NG PAM site in plants. xCas9 would be better to use as a highly specific SpCas9 in plant cells. Base editing with Cas9 variants has also been performed. SpCas9-NG, and SpCas9-VQR have successfully been applied in plant base editing [53, 61, 64, 65, 67, 69].

**Table 2:** Cas9 orthologs and engineered Cas9 variants

Cas9 Nuclease	Origin	PAM	Notes	References	Application to plant genome editing
SpCas9	*S. pyogenes*	NGG		See reviews ex. [1, 3, 8]	+
NmCas9	*N. meningitidis*	NNNNGMTT		[50]	-
StCas9	*S. thermophilus*	NNAGAAW		[51, 52]	+
SaCas9	*S. aureus*	NNGRRT, NNNRRT		[52–54]	+
CjCas9	*C.r jejuni*	NNNNRYAC		[55]	-
FnCas9	*F. novicida*	No		[56, 57]	+
SpCas9-VQR	*S. pyogenes*	NGA	Altered PAM	[58–61]	+
SpCas9-EQR	*S. pyogenes*	NGAG	Altered PAM	[58, 62]	+
SpCas9-VRER	*S. pyogenes*	NGCG	Altered PAM	[58, 59]	+
SpCas9-NG	*S. pyogenes*	NG	Altered PAM	[63–69]	+
SpCas9-HF1	*S. pyogenes*	NGG	High fidelity	[70, 71]	+
eSpCas9	*S. pyogenes*	NGG	High fidelity	[71, 72]	+
HypaCas9	*S. pyogenes*	NGG	High fidelity	[73]	-
evoCas9	*S. pyogenes*	NGG	High fidelity	[74]	-
Sniper-Cas9	*S. pyogenes*	NGG	High fidelity	[75]	-
xCas9	*S. pyogenes*	NG, GAA, GAT	Altered PAM, high fidelity	[65–68, 76–78]	+

Cas9 orthologs with different PAM preferences have been discovered in other bacteria [78], e.g., NmCas9 from *Neisseria meningitidis* [50], SaCas9 from *Staphylococcus aureus* [54], StCas9 from *Streptococcus thermophilus* [51], FnCas9 from *Francisella novicida* [56], and CjCas9 from *Campylobacter jejuni* [55]*.* Genes encoding most of these proteins are smaller than *SpCas9,* which is an advantage for gene delivery by viral vectors. FnCas9, StCas9, and SaCas9 have already been applied for the genome editing of *Arabidopsis* and tobacco [52, 57, 79]. Interestingly, Steinert et al. [52] demonstrated that the SaCas9 and SpCas9 systems do not interfere with each other in *Arabidopsis*. Such simultaneous targeting by Cas9 orthologs would enable multiplex genome engineering by targeting sites with different PAM sequences simultaneously, expanding the applicability of the CRISPR/Cas system. These Cas9 orthologs can be used for genome-editing application that until now involved SpCas9. For examples, SaCas9 was fused with base editors and successfully used for the base editing in rice [53].

Finally, new types of Cas proteins have been discovered recently that could potentially be applied to plant genome editing. For example, Cas13 (C2c2) protein belongs to a type VI CRISPR/Cas system that recognizes RNA sequences and exhibits RNA genome-editing activity [80, 81]. RNA editing is an alterative promising strategy for modifying the plant traits because RNA sequences can be modified without altering the genome sequence. The CRISPR/Cas13 system has been successfully applied in the targeted knockdown of endogenous genes in rice and *Nicotiana benthamiana* [80, 82]. Aman et al. [82] use the CRISPR/Cas13 system to engineer interference with RNA virus in *Arabidopsis*, suggesting that the system can be applied to engineer RNA-guided immunity against RNA virus in plants. As another example, Yan et al., [83] indicated the functional diversity of Type V CRISPR/Cas system (Cas12c, Cas12g, Cas12h, and Cas12i). Their functions range from dsDNA nicking and cleavage activity to the collateral cleavage activity of ssRNA and ssDNA, suggesting that the undiscovered Cas proteins with various functions still exist in nature. In addition, new types of CRISPR/Cas systems have also been isolated from uncultivated microbes [84]. For example, CRISPR/Cas14 system, classified as Type V CRIPSR/Cas system, can target and cleave ssDNA without PAM sequences [85]. CasX and CasY (classified into Cas12e, Cas12d, respectively) have unique structures, distinct from those of known Cas proteins, and CasX activity in genome editing has been validated in *E. coli* and human cells [86]. Based on its smallness, unique structure and unique DNA cleavage mechanism, CasX is expected to offer advantages relative to other genome-editing technologies. Finally, Cas3, a nuclease from *Thermobifida fusca* [87] and *E. coli* [88] Type I-E CRISPR/Cas system, induced large deletions, up to 100 kb, upstream of a target site in human cells. This unique characteristic would be useful for creating gene knockouts by causing deletions. Collectively, the application of these Cas proteins will expand the repertoire of plant genome-editing tools in the future.

## Conclusions

The emergence of genome-editing technologies has revolutionized plant genome engineering. In particular, the CRISPR/Cas9 system has accelerated the speed of research projects by providing easy, efficient, and precise approach to genome editing. Zsögön et al., [7] have successfully mimicked tomato domestication by genome editing, demonstrating the power of genome editing technology. In addition, CRISPR/Cas9 is no longer just scissors for cleaving the genome DNA. It can change one nucleotide into another and modify the epigenetic environments at the target site. The toolbox continues to be updated with newly developed CRISPR/Cas systems, Cas proteins and effector modules, etc. The preferred use of these tools varies depending on the research objective because they have different advantages and disadvantages, as discussed in this review.

Prime editing, developed recently in yeast and mammalian cells, is a promising technology for more precise editing of plant genomes. Interestingly, Anzalone et al., [22] succeeded in the knock-in of DNA fragments at the target site without donor DNA, thus not via the HDR pathway. This could be applied to plant genome editing because the knock-in of DNA fragments is difficult in plant cells. Prime editing would open new directions in plant genome editing.

On the other hand, the controlled induction of large deletions also represents a new strategy for gene knockout, deletion of gene clusters, and induction of chromosomal deletions. The Type I CRISPR system has been harnessed recently for genome engineering in human cells [87, 88] but not yet for plant genome engineering. It is also interesting to study what characteristics the Type I CRISPR system will show in plant cells.

Recently, several studies have also given a clue to solving long-standing problems with plant transformation systems. Plant transformation generally requires tissue culture, which is labor intensive, time-consuming and can be applied to only a limited number of plant species. It also runs the risk of inducing unintended somatic mutations during the regeneration process. To overcome these problems, *in planta* transformation systems that can avoid the tissue culture process have been reported recently [89, 90]. These will contribute to increasing the number of plant species that can be genome-edited and speed up plant genome editing studies.Precise genome editing is also advancing toward the removal of unnecessary DNA sequences and transgenes from the genome-edited plants. The *Cas9* gene is undesired in genome-edited plants because it could possibly induce mutations at off-target sites and also cause mosaicism in plants. The integration of a selection marker gene into unintended positions in the genome is also not wanted. In addition, the removal of unnecessary transgenes is required to alleviate regulatory concerns in some countries. Based on these factors, many researchers are trying to establish approaches for the efficient construction of null-segregants. As for now, targeted mutagenesis using RNPs is the best known strategy that could be used to achieve this purpose. However, further improvements will be necessary because the number of plant species to which this strategy can be applied is limited so far. In addition, the prime editing has a possibility that it could be used to achieve precise DNA-free genome editing because it can edit DNA sequences precisely without donor DNA templates. The application of prime editing technology to plant engineering has been expected.

Newly discovered CRISPR/Cas systems and the development of new technologies are being continuously reported, suggesting that the CRISPR toolbox for plant engineering will expand further in the near future. This set of tools will provide new approaches to achieve precise genome editing without any traces of transgenes remaining in genome-edited plants.

## Data Availability

Not applicable.

## Abbreviations

ZFN: Zinc finger nuclease
TALEN: Transcription activator-like effector nuclease
CRISPR/Cas9: Clustered regularly-interspaced short palindromic repeats/Cas9
Cas9: CRISPR-associated protein 9 nuclease
DSB: Double-strand break
NHEJ: Nonhomologous end-joining
HDR: Homology-directed repair
indel: insertion and deletion
gRNA: guide RNA
PAM: Protospacer adjacent motif
SpCas9: *Streptococcus pyogenes* Cas9
GT: Gene targeting
dCas9: dead Cas9
BE: Base editor
CBE: Cytidine deaminase-based DNA base editor
AID: Activation-induced cytidine deaminase
ABE: Adenine deaminase-based DNA editor
ALS: Acetolactate synthase
ACCase: Acetyl-coenzyme A carboxylase
IVT: In vitro transcript
RNP: Ribonucleoprotein
